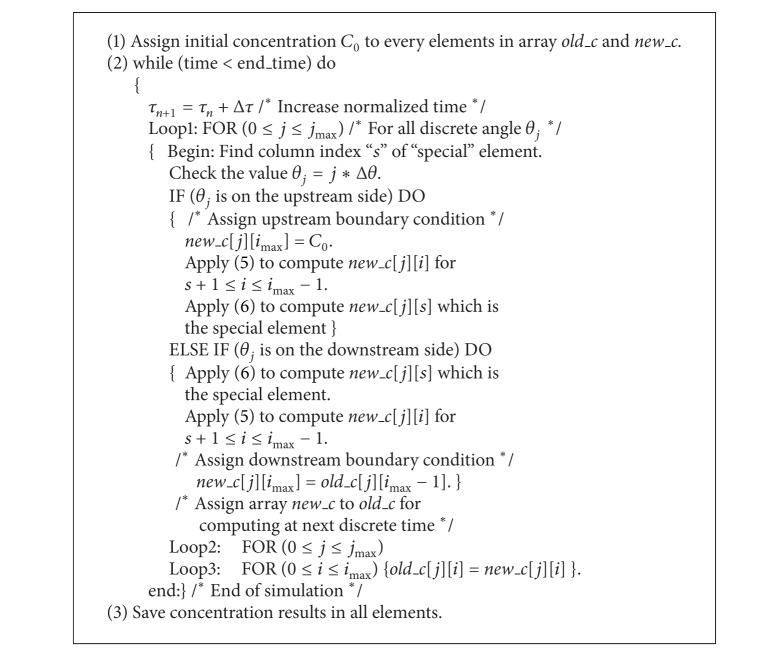# Parallel Simulation of HGMS of Weakly Magnetic Nanoparticles in Irrotational Flow of Inviscid Fluid

**DOI:** 10.1155/2014/519654

**Published:** 2014-05-11

**Authors:** Kanok Hournkumnuard, Banpot Dolwithayakul, Chantana Chantrapornchai

**Affiliations:** ^1^Department of Physics, Faculty of Science, Silpakorn University, Nakhon Pathom 73000, Thailand; ^2^Department of Computing, Faculty of Science, Silpakorn University, Nakhon Pathom 73000, Thailand; ^3^Department of Computer Engineering, Faculty of Engineering, Kasetsart University, Bangkok 10900, Thailand

## Abstract

The process of high gradient magnetic separation (HGMS) using a microferromagnetic wire for capturing weakly magnetic nanoparticles in the irrotational flow of inviscid fluid is simulated by using parallel algorithm developed based on openMP. The two-dimensional problem of particle transport under the influences of magnetic force and fluid flow is considered in an annular domain surrounding the wire with inner radius equal to that of the wire and outer radius equal to various multiples of wire radius. The differential equations governing particle transport are solved numerically as an initial and boundary values problem by using the finite-difference method. Concentration distribution of the particles around the wire is investigated and compared with some previously reported results and shows the good agreement between them. The results show the feasibility of accumulating weakly magnetic nanoparticles in specific regions on the wire surface which is useful for applications in biomedical and environmental works. The speedup of parallel simulation ranges from 1.8 to 21 depending on the number of threads and the domain problem size as well as the number of iterations. With the nature of computing in the application and current multicore technology, it is observed that 4–8 threads are sufficient to obtain the optimized speedup.

## 1. Introduction

High gradient magnetic separation (HGMS) is a separation technique which has been proven as a powerful one for the capture of weakly magnetic particles from suspension which conventional magnetic separation techniques using only permanent magnet cannot achieve. Some examples of potential applications of HGMS technique in the fields of biomedical and environmental science are the separation of red and white blood cells from small amount of blood sample in microfluidic device [1], trapping of infected red blood cell [2], and the separation of specific inorganic compounds from inland water [3]. Recently, many research works [4–6] have been studied to investigate the feasibility of using the principle of HGMS for concentrating nanotherapeutic carriers in specific regions within living body. This technique is called magnetic drug targeting (MDT). It is seen that the technique of HGMS becomes that which plays important role in nanotechnology so studying to understand the mechanism of nanoparticles capture by HGMS technique in various situations becomes useful for various research areas.

In this work, we study the problem of HGMS of weakly magnetic particle using a microferromagnetic wire as capture center in irrotational flow of inviscid fluid. The effect of diffusion becomes significant for nanoparticle motion so dynamic of capture process will be described statistically in terms of particle volume concentration. The problem is considered in an annular domain surrounding the wire. The continuity equation describing time rate of change of volume concentration in any part of the domain is formulated and is solved numerically as an initial and boundary value problem by using the explicit finite-difference method. A parallel algorithm for updating concentration value at each time step is developed based on openMP (http://openmp.org/wp/) to speed up the simulation because time step must be sufficiently small for the explicit method to obtain accurate simulation results.

## 2. Problem Description and Backgrounds

**Figure 1:**
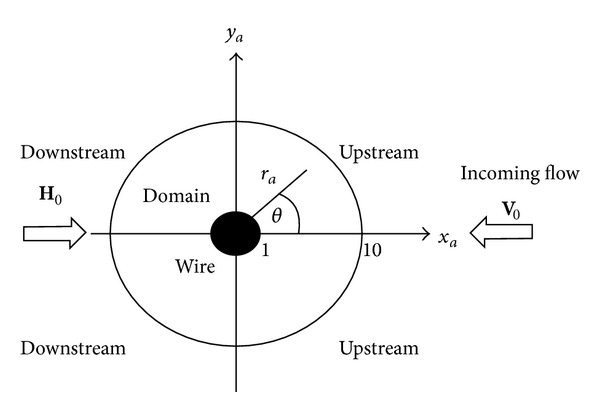
The simulation domain, direction of incoming fluid flow (**V**_0_), applied uniform magnetic field (**H**_0_), and the coordinates systems (not to scale).

Figure 1 illustrates the definition of the problem. A long cylindrical ferromagnetic wire of radius *a* is placed traverse a flow of fluid containing weakly magnetic particles. In Figure 1, the flow is parallel to *xy* plane and wire axis is parallel to *z*-axis normally pointing out of the page. The distance is measured in the unit of wire radius. A uniform external magnetic field is applied perpendicular to wire axis and opposite to the incoming flow direction. This configuration is called the longitudinal mode operation. In Figure 1, the half of the domain on the right hand side of *y*-axis is called the upstream side while another half is called downstream side. For long wire, the flow profile of the fluid varies only on the *xy* plane but does not depend on *z* coordinates [7]. Moreover, the high gradient magnetic field around the wire also varies only on the *xy* plane [7]. Consequently, the problem can be treated as a two-dimensional one and the simulation domain is set up as an annular region surrounding the wire with the inner radius equal to wire radius and the outer radius is multiples of wire radius where the gradient of magnetic field is almost zero.

### 2.1. The Magnetic Field and Magnetic Force

By solving a magnetostatic boundary value problem in the domain using the boundary condition of uniform magnetic field (*H*_0_) in the region that is far from the wire and magnetostatic boundary condition at the surface of the wire, the high gradient magnetic field (**H**) around the wire can be expressed in polar coordinates (*r*_*a*_, *θ*) as [7]

(1) $$\begin{array}{c} H=H_{0}\left[\left(1+\frac{K_{W}}{r_{a}^{2}}\right)\cos\theta r-\left(1-\frac{K_{W}}{r_{a}^{2}}\right)\sin\theta \theta \right], \end{array}$$

where *K*_*W*_ = *M*/2*H*_0_ is the demagnetization factor of the wire, *M* is the magnetization of the wire, and the boldface symbols **r** and *θ* represent the unit vector in radial and plane angular directions, respectively. The symbol *r*_*a*_ = *r*/*a* means the radial distance in the unit of wire radius *a*. In the regions of high gradient magnetic field near to wire surface, the local magnetic field strength *H* is higher than the externally applied uniform magnetic field *H*_0_, hence increasing the magnetic force acting on any weakly magnetic particles in these regions.

The magnetic force acting of any weakly magnetic particle of susceptibility *χ*_*p*_ dispersed in a fluid medium of susceptibility *χ*_*f*_ satisfied the equation [7]

(2) $$\begin{array}{c} F_{m}=\mu_{0}\left(\chi_{p}-\chi_{f}\right)V_{p}H\nabla H, \end{array}$$

where *μ*_0_ = 4*π* × 10^−7^ henry/m is the magnetic permeability of free space, *V*_*p*_ is volume of a particle, and the operator ∇ expresses the gradient or nonuniformity of local magnetic field strength *H*.

### 2.2. Fluid Flow Field

In this work, we follow the work of Davies and Gerber [8] by considering an irrotational flow of inviscid fluid around the wire. The radial (*V*_*r*_) and angular (*V*_*θ*_) components of fluid in the domain can be expressed as [7]

(3) $$\begin{array}{c} V_{r}=V_{0}\left(1-\frac{1}{r_{a}^{2}}\right)\cos\theta ,\\ V_{\theta }=-V_{0}\left(1+\frac{1}{r_{a}^{2}}\right)\sin\theta , \end{array}$$

where *V*_0_ is the incoming speed of fluid at the outer boundary of the domain.

### 2.3. The Continuity Equation

Dynamic of nanoparticle concentration in the domain is described by the continuity equation which states that, in any small elements of the domain, time rate of change of particle volume concentration is proportional to net volume flux passing through the elements and can be expressed as

(4) $$\begin{array}{c} \frac{\partial C}{\partial t}=D\nabla^{2}C-\nabla \cdot \left(CV_{B}\right)-\nabla \cdot \left(\frac{DCF_{m}}{k_{B}T}\right), \end{array}$$

where *C* is particle volume concentration, *t* is time, *D* is diffusion coefficient, **V**_*B*_ is fluid velocity, *k*_*B*_ is Boltzmann's constant, and *T* is absolute temperature in Kelvin. The first, second, and third terms on the right hand side represent particle volume flux caused by diffusion process, convection by fluid flow, and the competition between effects of magnetic force and diffusion, respectively. Equation (4) is solved numerically, by using the explicit finite-difference method, as initial and boundary values problems to determine the pattern of concentration distribution within the domain.

## 3. Simulation Methodology

**Figure 2:**
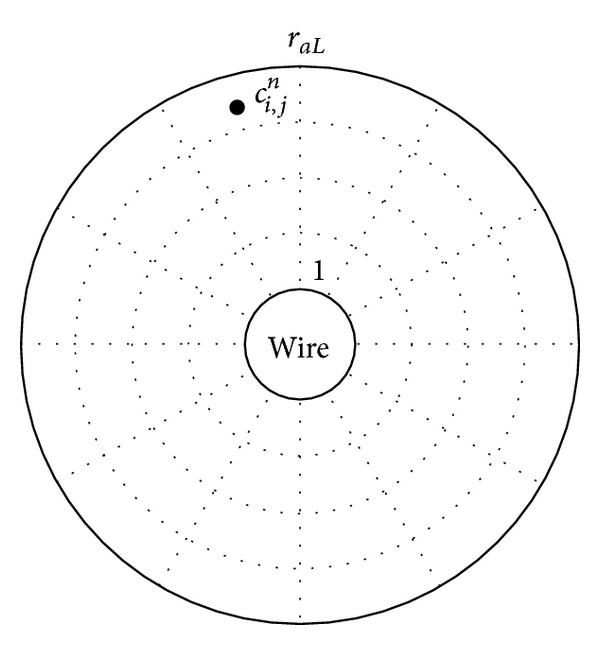
Simulation domain partitioning.

We partition the annular domain in Figure 1 into a structured mesh using normalized radial and angular steps Δ*r*_*a*_ and Δ*θ*, respectively, as shown in Figure 2. At wire surface *r*_*a*_ = 1 and at outer boundary *r*_*a*_ = *r*_*aL*_ = 10. The symbol *C*_*i*,*j*_^*n*^ represents the volume concentration at the middle point (*r*_*a*,*i*_, *θ*_*j*_) of each element at an instantaneous time *t*_*n*_.

In Figure 2, there exist two types of domain element. The first type is “ordinary” element where particle flux can pass through every surface and the second type is called “special” element which at least one of its surfaces is in contact with wire surface or the surface of static accumulation of particle. To solve (4), all derivative operations are approximated by using the finite-difference relations. The difference equation for “ordinary” element can be expressed as

(5) Ci,jn+1−Ci,jnΔτ =(Ci+1,jn−2Ci,jn+Ci−1,jn(Δra)2)+1(ra)i(Ci+1,jn−Ci−1,jn2(Δra))  +1(ra)i2(Ci,j+1n−2Ci,jn+Ci,j−1n(Δθ)2)−(Gr)i,jCi,jn(ra)i  −(Gr)i,j(Ci+1,jn−Ci−1,jn2(Δra))−(∂Gr∂ra)i,jCi,jn  −(Gθ)i,j(ra)i(Ci,j+1n−Ci,j−1n2(Δθ))−Ci,jn(ra)i(∂Gθ∂θ)i,j.

For “special” element, the corresponding difference equation has the form as

(6) Cs,jn+1−Cs,jnΔτ=1(ra)s2(Cs,j+1n−2Cs,jn+Cs,j−1n(Δθ)2) −(Gθ)i,j(ra)s(Cs,j+1n−Cs,j−1n2(Δθ))−Cs,jn(ra)s(∂Gθ∂θ)s,j +(Gr)s,jCs,jnΔra−(Cs+1,jn−Cs,jnΔra),

where the functions *G*_*r*_ = (*aV*_*r*_/*D*)+(*aF*_*mr*_/*k*_*B*_ *T*) and *G*_*θ*_ = (*aV*_*θ*_/*D*)+(*aF*_*mθ*_/*k*_*B*_ *T*) and *τ* = *Dt*/*a* ^2^ is the normalized time.

### 3.1. Simulation Algorithm

**Figure 3:**
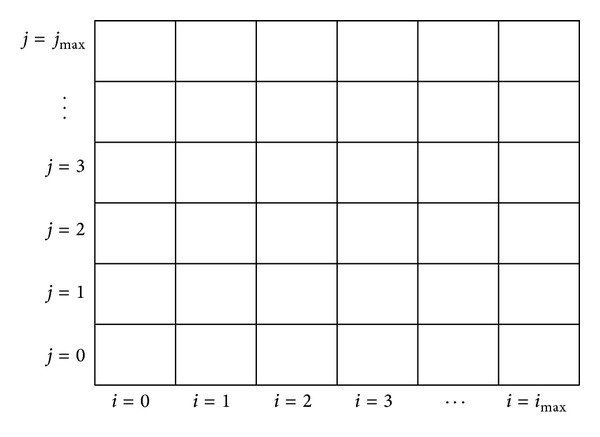
The concentration array.

In the simulation, two two-dimensional arrays of equal size named *old*_*c* and *new*_*c* are declared for saving the concentrations *C*_*i*,*j*_^*n*^ and *C*_*i*,*j*_^*n*+1^ at the old and new instantaneous times *t*_*n*_ and *t*_*n*+1_, respectively. The numbers of row and column of each array are 360/Δ*θ* and [(*r*_*aL*_ − 1)/Δ*r*_*a*_] + 1, respectively.  Figure 3 shows a concentration array of maximal column and row indices as *i*_max⁡_ and *j*_max⁡_, respectively, where *i*_max⁡_ = total column – 1 and *j*_max⁡_ = total row – 1.

The steps of simulation can be summarized as shown in Algorithm 1.

The size of normalized time step Δ*τ* must be small enough to provide stable numerical results. The size of Δ*τ* in this work is assigned to satisfy Courant stability condition as follows:

(7) $$\begin{array}{c} \Delta \tau \leq \frac{1}{G_{\max}\sqrt{1/{\left(\Delta r_{a}\right)}^{2}+1/{\left(\pi *\Delta \theta /180\right)}^{2}}}, \end{array}$$

where *G*_max⁡_ is the maximal magnitude of *G*_*r*_ and *G*_*θ*_ which is spatial function but time independent. When stable condition (7) is satisfied, the order of error of computed concentration is [(Δ*τ*/2)+(Δ*r*_*a*_)^2^/12 + (*π*∗Δ*θ*/180)^2^/12].

### 3.2. Parallel Approach

In this work, we use openMP platform to parallelize the above algorithm. To simplify this, the challenge of the algorithm is twofold.

1. The domain size can be large due to the domain size of Δ*r*_*a*_ and Δ*θ*. If Δ*θ* = 0.0087 radian or 0.5 degree, we obtain that the number of rows for array *C* is 720. For various domain size and Δ*r*_*a*_, the number of columns for array *C* can be large. Thus, this is a big piece of shared memory considering openMP approach.
2. The number of iterations is more due to Δ*τ*. The value should be small enough so that the simulation result is stable. When the number of iterations is large, it could take hours to days to finish the simulation.

Due to the first issue, there is no dependency in computing each element in array *C*. We use double buffer to store the current *C* (*new*_*c*) and the *C* in the previous iteration (*old*_*c*). Each element *C*_*i*,*j*_ can be computed independently by (5)-(6). Thus, for simplicity, we divide the work sharing using row-wise method. Each thread works on rows of computing *new*_*c*[*i*][*j*].

Depending on the openMP schedule used, the work rows may be distributed to threads statically or dynamically. For example, we may try with the clause* static* with *chunk*_*size* = *N*/*T* where *N* is the total number of rows and *T* is the number of threads which assigns the work statically. Or we may try with the clause* static* with *chunk*_*size* = 1 where the row number is assigned to the thread *N mod T* statically. Similarly, the clause* dynamic* with *chunk*_*size* = 1. One row is assigned to compute by the thread and when the thread finishes, it will compute on the next row. The row assignment is done dynamically. Each thread may not get the same number of rows to compute on.

**Figure 4:**
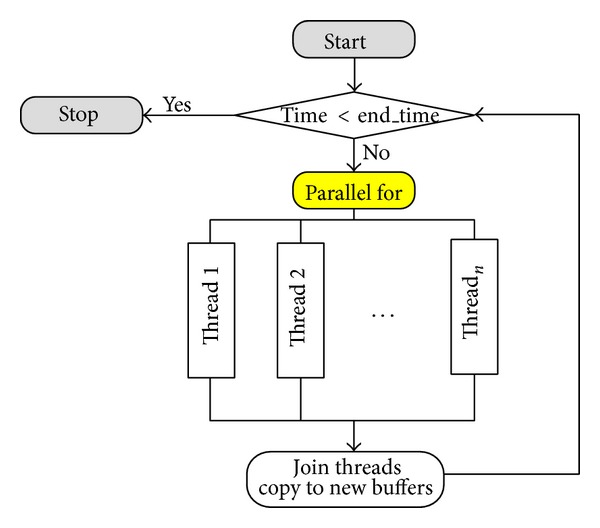
Threads are created based on each angle.

The algorithm for computing the simulation is similar to the above one except that the line marked by Loop1 is the* parallel for*. Also, all the terms are private variables except the concentration arrays (*new*_*c* and *old*_*c*).  Figure 4 presents how we fork the parallel threads. The Loop1 corresponds to the* parallel for*. Each thread computes the rows it is responsible for based on the schedule approach. That is, it performs the code in line “Begin” until line “End” in the algorithm. After all the threads finish, the *new*_*c* is copied and they can proceed to the next time step.

## 4. Results

We present the results in two parts. The first part is the results of the correctness of the simulation approach and the second part is the performance of the parallel computation on the varying factors such as the domain size and the iteration numbers.

### 4.1. Simulation Results

In this work, we simulate concentration dynamics of weakly magnetic particle which has known magnetic susceptibility value. Simulation parameters are obtained from the previously reported work of Davies and Gerber [8]. We consider manganese pyrophosphate (Mn_2_P_2_O_7_·3H_2_O) particles of 24 nm diameter dispersed in water that provides *χ*_*p*_ − *χ*_*f*_ = +2.03 × 10^−3^. The ferromagnetic wire has 50 micron radius and magnetization of 8.61 × 10^5^ A/m. Water with volume concentration *C*_0_ = 0.0010 of Mn_2_P_2_O_7_·3H_2_O particle flows traverse to the wire with incoming velocity of 1 × 10^−5^ m/s. The external uniform magnetic field *H*_0_ = 1 × 10^7^ A/m. The saturation or maximal volume concentration in any element is limited at *C*_sat⁡_ = 0.10. The radial and angular steps for partitioning the simulation domain are Δ*r*_*a*_ = 0.010 and Δ*θ* = 0.0087 radian or 0.5 degree. The normalized time step is Δ*τ* = 1 × 10^−5^ which is small enough to obtain stable results. The normalized radius at the outer boundary, *r*_*aL*_, which indicates domain size, is varied between 3, 5, and 9. From the value of Δ*r*_*a*_ and Δ*θ*, the number of rows in concentration array is 720 and the numbers of columns are 201, 401, and 801. The maximal row index is *j*_max⁡_ = 719 while the maximal column indices are *i*_max⁡_ = 200, 400, and 800 for each case.

**Figure 5:**
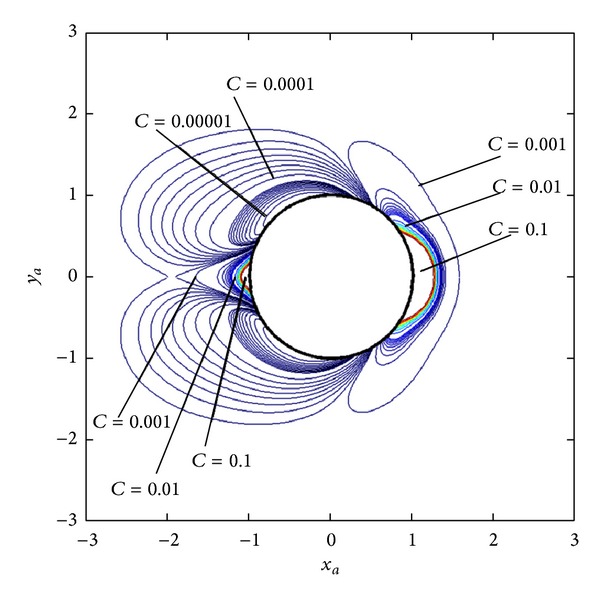
The concentration contours of Mn_2_P_2_O_7_·3H_2_O nanoparticles of 24 nm diameter at *t* = 10.24 s.

Figure 5 shows the contours of particle volume concentration at time *t* = 10.24 s which is obtained after 7500 rounds of concentration updating. The figure shows the contours only within the area of *r*_*a*_ ≤ 3.0 because the concentration in farther region is almost smooth at *C* = *C*_0_ = 0.001 and no significant concentration gradient is observed. The pattern of the contours is compared with previously reported results of Davies and Gerber [8] using *r*_*aL*_ = 3.0 and we found similar pattern of concentration contours. In Figure 4, the saturation concentration *C*_sat⁡_ = 0.1 where particles accumulate densely exists in the horizontal direction parallel to the directions of **H**_0_ and **V**_0_ indicated in Figure 1. Consequently, particle concentration can be increased 100 times from 0.001 to 0.1. The result shows that HGMS technique can concentrate even weakly magnetic nanoparticles. Consequently, it is feasible to increase the concentration of other weakly magnetic nanoparticles with lower magnetic susceptibility value. Some examples of these particles are nanodrug carriers with gold core which are synthesized for using in cancers and tumors therapies nowadays. The magnetic susceptibility of gold particle larger than 10 nm is in the order of 10^−5^ which is about two orders of magnitude lower than that of Mn_2_P_2_O_7_·3H_2_O in this work so the concentration of gold nanodrug carriers may not be increased 100 times. However, increasing of drug carriers concentration from only 2 to 4 times is sufficient for therapy process so HGMS is very a interesting technique to increase the effectiveness of cancers or tumors therapies. It is also observed that the region of accumulation of the particles, *C* > *C*_0_, occurs within the region of *r*_*a*_ ≤ 2.0 which is very close to wire surface. This means that particles dispersed outside of this region are not captured on the wire. Consequently, capture efficiency can be increased by using many wires arranged in some configurations with distance between wires about 2 times of their radius.

**Figure 6:**
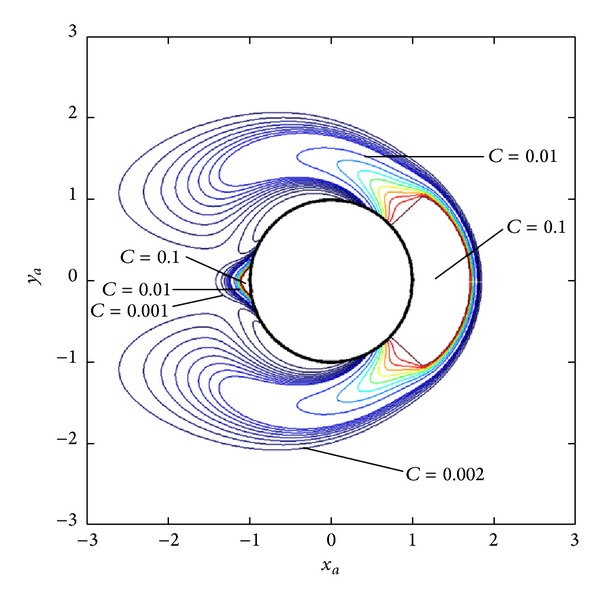
The concentration contours of Mn_2_P_2_O_7_·3H_2_O nanoparticles of 24 nm diameter at *t* = 34.12 s.

Figure 6 shows the contours of particle volume concentration at time *t* = 34.12 s which is obtained after 25,000 rounds of concentration updating. The saturation region on the upstream side is larger while no significant change of saturation region on the downstream side is observed. The pattern of simulated particle accumulation is in good agreement with the experimental results collected in the book of Gerber and Birss [7].

### 4.2. Performance Results

In the experiments, we test the simulation time on Intel Xeon Phi 7110P located at Kasetsart University, Thailand. It has 61 cores at 1.1 GHz, with 64-bit addressing running Linux 2.6* and gcc 4.4.7. It contains the same two MICs. We test the simulation time based on the two issues above: the domain size and the total iterations. The domain size is varied in the maximal column indices as *i*_max⁡_ = 200, 400, and 800 for each case. The maximal row index is *j*_max⁡_ = 719. The numbers of iterations tested are 5,000 and 10,000. In the experiments, we have tried more numbers of iterations but the results are quite stable when the iteration is closed to 10,000.

**Figure 7:**
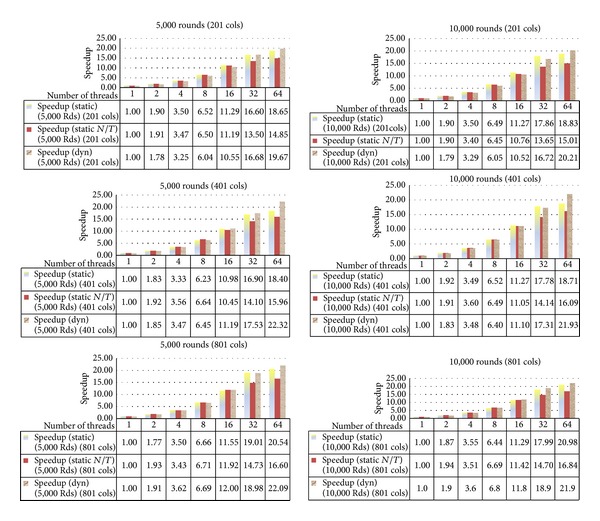
The speedup for the varying number of columns, 201, 401, and 801, and varying number of iterations, 5,000 and 10,000 iterations, varying schedule types.

Figure 7 compares varying schedule types of openMP in varying the domain sizes and the iteration numbers. We test for static schedule with *chunk*_*size* = 1 (static), static schedule with *chunk*_*size* = *N*/*T* (static *N*/*T*), and dynamic schedule with *chunk*_*size* = 1 (dynamic). It is clear that the dynamic schedule outperforms the static ones in every case. This is because the work on each row is not the same. For some angle, the number of points to compute may decrease when the numbers of iterations are more. The density of the concentration is less when the location is farther away from the core. It is noted that when we double the number of threads, we obtain almost double speedup for each case until 16 threads. When the number of threads is 32, or more, the speedup is reduced since there may be too many threads for the number of rows (720). Then, the thread overhead is more. We obtain less speedup.

**Figure 8:**
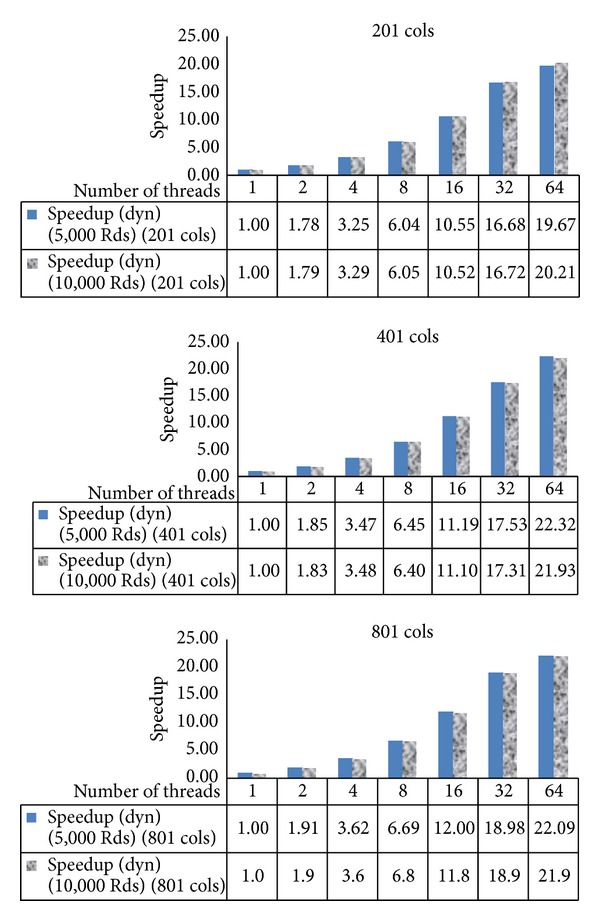
The speedup for the case number of columns, 201, 401, and 801 with the dynamic schedule varying number of iterations, 5,000 and 10,000 iterations.

For 5,000 iterations, 1 thread, 98,585 milliseconds is used while for 10,000 iterations, 1 thread, 195,396 milliseconds is used. From Figure 8, when we double the number of iterations, the speedup goes in the same manner when doubling the number of threads with the dynamic schedule although perhaps some case like 32 and 64 threads yields a little bit more speedup.

**Figure 9:**
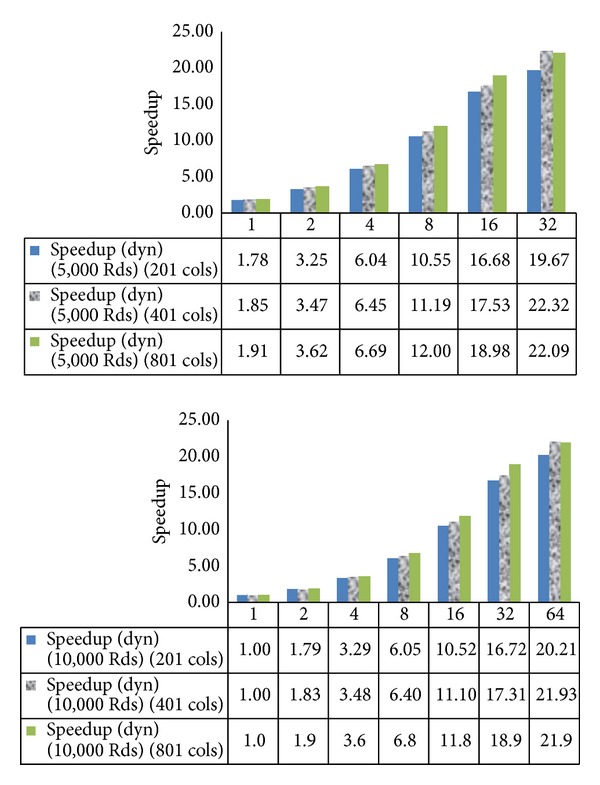
The speedup for 5,000 and 10,000 iterations, dynamic schedule varying number of columns.

Figure 9 presents the speedup for 5,000 and 10,000 iterations when increasing the domain size by the double. Again, the approach still gives the similar speedup when the domain size is double from 201 to 401 and from 401 to 801 when the number of threads is double.

**Figure 10:**
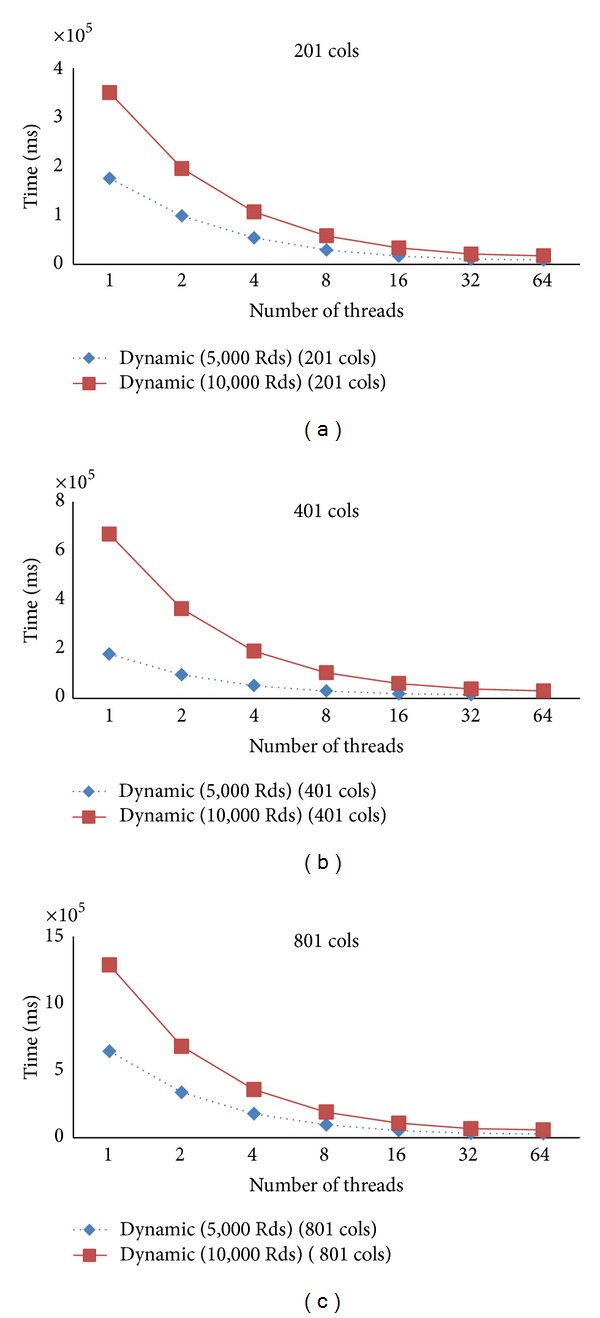
The computation time for the case numbers of columns, 201, 401, and 801, dynamic schedule varying number of iterations.

**Figure 11:**
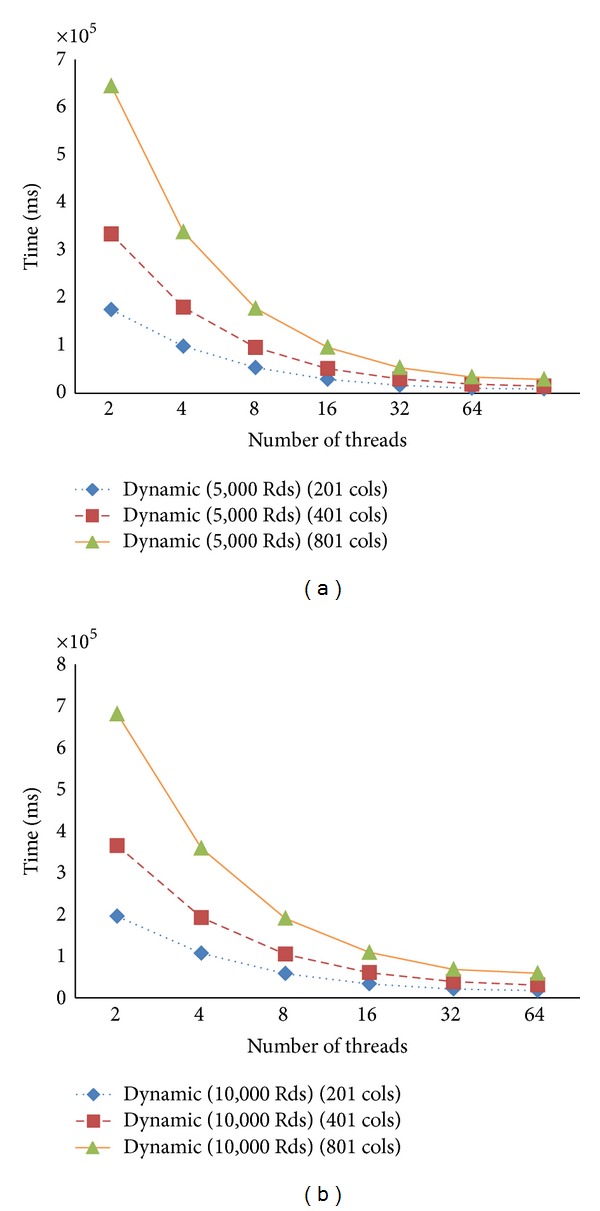
The computation time for 5,000 and 10,000 iterations, dynamic schedule varying number of columns.

Figure 10 presents the computation time for the 5,000 and 10,000 iterations. It shows the trends of the reduction of the computation time for each case. Similarly, in Figure 11, we show the trend of the reduction in computation when the domain size is double.

**Figure 12:**
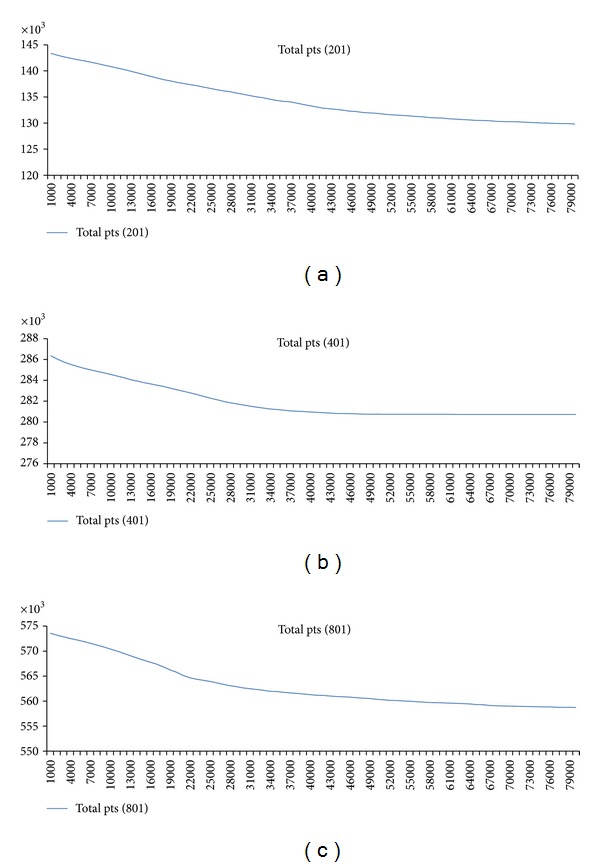
Total points calculated for every 1,000 iterations for the case of 201, 401, 801 column size.

**Figure 13:**
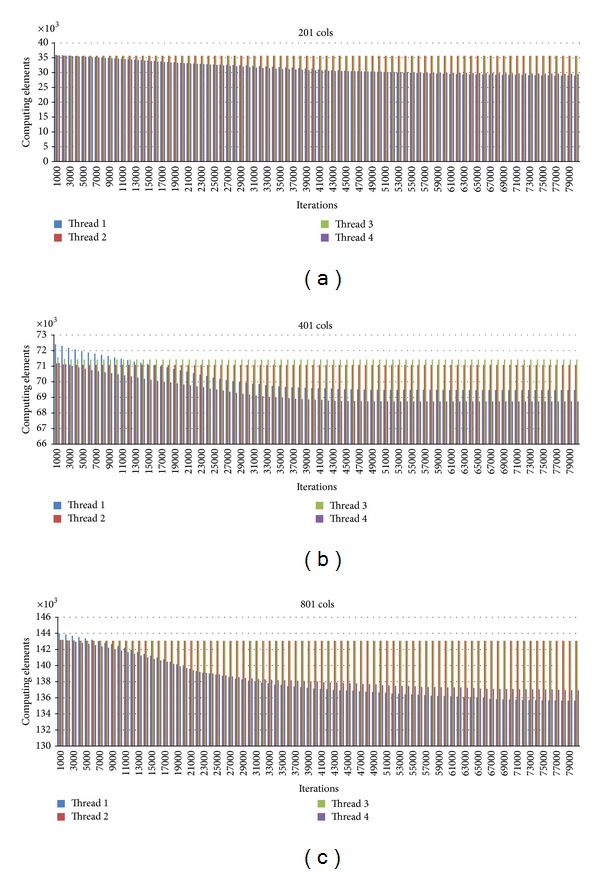
The workload of each thread for 4 threads using static schedule *chunk*_*size* = *N*/*T*.

**Figure 14:**
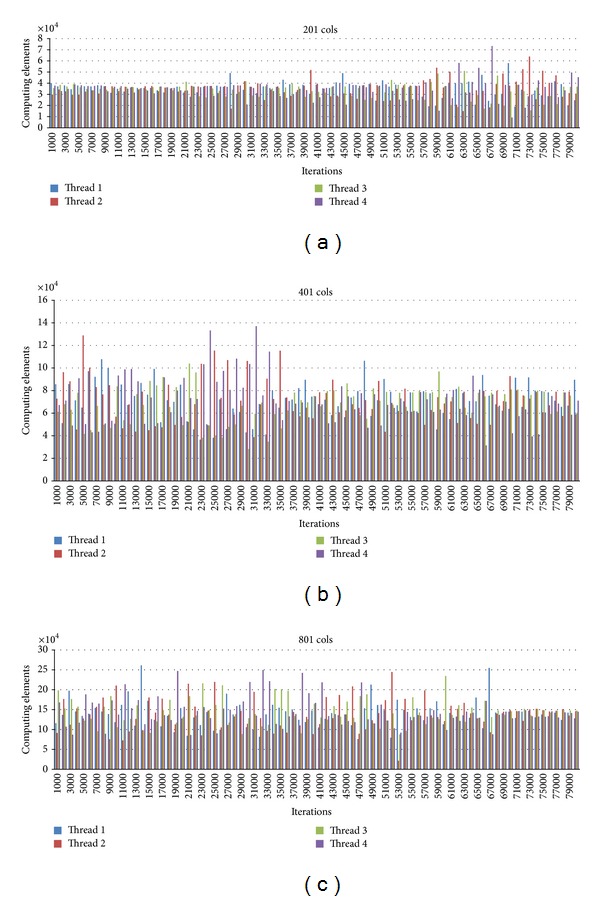
The workload of each thread for 4 threads using dynamic schedule *chunk*_*size* = 1.

For further analysis, we study the workload for each thread at each iteration. We observe that the number of points calculated for each thread is very much stable for every 1,000 iterations. For the first 1,000 iterations, all threads have about the same amount of points calculated. As the iteration goes, the concentration values become steady at some points. Thus, the thread responsible for the rows containing more of these points has less work load. Therefore the static schedule is not best performed. With the dynamic schedule, the thread that finishes fast due to this reason will be able to perform computation in other rows. Figures 12–14 show graphs related to the issues.

Figure 12 shows how the total elements needed to be calculated are reduced as the iterations pass by. We show this for 80,000 iterations. It is seen that the number of points is gradually reduced. In 80,000 iterations, about 10,000 points are reduced for every case.

Figure 13 presents the workload for static schedule using *chunk*_*size* equal to the number of rows divided by the number of threads. These examples are for 4 threads. It is seen that the 2nd and 3rd threads have more points to be calculated since the works are divided by rows. This is because the special points are more in Quadrants 1 and 4. Compare to Figure 14. With the dynamic schedule, the threads obtain the row as it finishes. The workload distribution is quite random. With this nature of steady points, the task division by other methods such as column-wise or block-wise may also be investigated in the future.

## 5. Conclusions

In this work, we present the process of high gradient magnetic separation (HGMS) using microferromagnetic wire for capturing the weakly magnetic nanoparticles in the irrotational flow of inviscid fluid. The parallel simulation using openMP is proposed. The domain problem is viewed in two-dimensional domains of wire radius and annular step and with the magnetic fore and fluid flow. The timing factors are the size of granularity and the iterations performed. Both also affect the correctness of the simulation process.

To parallelize it, each concentration element can be computed independently. Each thread computes rows of each concentration. The varying schedule types are considered. From the experiments, the dynamic schedule is very suitable since, along the iterations, the number of computed elements is less when the simulation moves gradually to the stable state. Also, with the parallelization by angle, we obtain double speedup when the number of threads goes double. This is consistent for the case when we double the domain size and double number of iterations. For the study of 720 rows, the number of threads should be 16 for the optimum speedup.

In the future, if the domain is very large, we can distribute the domain using MPI approach, and within each MPI node, the proposed openMP method can be used. With the MPI, the concentration array is divided with overlapped rows [9]. The synchronization is needed between iterations before going to the next iteration. The nonblocking communication may be considered to hide this latency [10].

## Figures and Tables

**Algorithm 1:**